# Specific IgG Antibodies React to Mimotopes of BK Polyomavirus, a Small DNA Tumor Virus, in Healthy Adult Sera

**DOI:** 10.3389/fimmu.2017.00236

**Published:** 2017-03-06

**Authors:** Silvia Pietrobon, Ilaria Bononi, Elisa Mazzoni, Francesca Lotito, Marco Manfrini, Andrea Puozzo, Federica Destro, Giovanni Guerra, Pier Francesco Nocini, Fernanda Martini, Mauro G. Tognon

**Affiliations:** ^1^Laboratories of Cell Biology and Molecular Genetics, Department of Morphology, Surgery and Experimental Medicine, Section of Pathology, Oncology and Experimental Biology, School of Medicine, University of Ferrara, Ferrara, Italy; ^2^Clinical Laboratory Analysis, University Hospital of Ferrara, Ferrara, Italy; ^3^Department of Surgery, Section of Oral and Maxillofacial Surgery, School of Medicine, University of Verona, Verona, Italy

**Keywords:** BK polyomavirus, immunology, serum, antigen, mimotope, enzyme-linked immunosorbent assay

## Abstract

BK polyomavirus (BKPyV) was isolated in 1971 from the urine of a kidney transplant patient. Soon after its identification, BKPyV was characterized as a kidney-tropic virus, which is responsible of a significant fraction of the rejection of transplant kidney in the host. Moreover, in experimental conditions, BKPyV is able to transform different types of animal and human cells and to induce tumors of different histotypes in experimental animals. BKPyV DNA sequences have been detected in healthy individuals and cancer patients using polymerase chain reaction/Shouthern blot hybridization methods. Serum antibodies against this polyomavirus were revealed using immunological techniques, which, however, cross-react with other polyomaviruses such as JC (JCPyV) and Simian Virus 40. These non-specific data indicate the need of novel immunological methods and new investigations to check in a specific manner, BKPyV spread in humans. To this aim, mimotopes from BKPyV structural capsid protein 1 (VP1) were employed for specific immunological reactions to IgG antibodies of human serum samples. An indirect enzyme-linked immunosorbent assay with synthetic peptides mimicking immunogenic epitopes of BKPyV VP1 was set up and employed to test sera of healthy adult subjects. Data from this innovative immunological assay indicate that serum antibodies against BKPyV VP1 mimotopes are detectable in healthy subjects ranging from 18 to 90 years old. The overall prevalence of serum samples that reacted to BKPyV VP1 mimotopes was 72%. The strong points from this investigation are the novelty of the immunological method, its simplicity of the approach, and the specificity of BKPyV antibody reaction to VP1 mimotopes.

## Introduction

BK polyomavirus (BKPyV) was isolated in 1971 by Gardner et al. from the urine of a renal transplant patient (1). Soon after its identification, BKPyV was characterized as a human polyomavirus (HPyV) with a circular double-stranded DNA of about 5.15 Kb (2). In experimental conditions, BKPyV is able to transform human cells of different types and to induce tumors of different histotypes in experimental animals (3).

In most cases, BKPyV primary infection occurs during childhood (2). However, some investigations shown that BKPyV could even be vertically transmitted from mother to embryo/fetus (4).

Many reports have indicated a high prevalence of serum IgG antibodies against BKPyV in different human populations worldwide (2, 5, 6), suggesting that BKPyV is a ubiquitous HPyV.

After primary infection, BKPyV remains in the human host lifelong as a latent/persistent infection. BKPyV may reactivate in immunocompromised patients or during immune depression. In bone marrow and kidney transplant patients, BKPyV infection may cause hemorrhagic cystitis and kidney rejection (7, 8).

Many studies have reported an association between BKPyV and diseases of the urinary, genital, or upper respiratory tracts. BKPyV footprints have been detected at high prevalence in cancers of different histotypes, such as brain, bone, prostate, insulinoma, Kaposi’s sarcomas, urinary, and genital tumors (3). In a 2012 meeting organized by the World Health Organization, held in Lyon, France, BKPyV was classified as “possibly carcinogenic to humans” (9).

BK polyomavirus encodes two viral oncogenes, the large T antigen (Tag) and small Tag (tag), which transform different types of animal and human cells. Specifically, Tag binds and abolishes the functions of tumor suppressor p53 and pRB family proteins. Tag is also clastogenic and mutagenic. Small tag interacts with phosphatase PP2A, which activates the Wnt pathway. Moreover, tag activates phosphatidylinositol 3-kinase, an enzyme involved in pathways crucial for cell proliferation and transformation. These Tag and tag activities are able to adversely affect the cellular genome where gene mutations may accumulate. Due to Tag binding, in the absence of p53 functions, the cellular DNA then remains unrepaired and consequently the genome “derails” (3, 10). In addition, it has been shown that, in prostate carcinoma (PCa), loss-of function mutations in the p53 gene at very early stages are rare. Therefore, the sequestration of wild-type p53 exerted by BKPyV Tag oncoprotein is considered a hallmark for BKPyV involvement, during initial phases (11). These mechanisms may ensure PCa genetic heterogeneity. However, at present, there are insufficient human epidemiological data to support this hypothesis (10). Considering that BKPyV infection is ubiquitous in the general population with a prevalence of serum antibodies against this polyomavirus of up to 90% worldwide (2, 5, 6), it is difficult to assess whether this virus has a specific role of in cellular transformation (12).

BK polyomavirus is the causal agent of polyomavirus-associated nephropathy. Indeed, up to 36% of kidney transplant patients are at risk of premature allograft failure (13). At-risk patients can be identified before significant functional impairment of the renal allograft occurs (13–17). For this reason, the study of more precise and advanced techniques detecting BKPyV infection and reactivation is important.

Taken overall, these data prompted us to develop and set up a new indirect enzyme-linked immunosorbent assay (ELISA) in order to determine BKPyV antibody and its titer. Until now, immunological methods mainly employed virus-like particles (VLPs)/recombinant viral protein (VP) 1 (VP1) as antigens. Data obtained using these approaches were always influenced by some cross-reactivity among the three polyomaviruses BKPyV, JCPyV, and Simian Virus 40 (SV40) (18). Indeed, BKPyV displays strong antigenic homology with JCPyV and SV40 (6, 19, 20). In this study, a specific sensitive innovative indirect ELISA with synthetic peptides was employed to test BKPyV spread in healthy adult subjects.

## Materials and Methods

### Human Samples

Serum samples (*n* = 446) were collected from healthy subjects (HSs), mean age = 49.73, at the Clinical Laboratory Analysis, University Hospital of Ferrara. Sera were taken from discarded laboratory analysis samples, after routine analyses at the University Hospital, Ferrara, Italy, before their destruction by incineration. The hospital records annotated these serum samples as belonging to HS. Indeed, parameters of blood analyses were all in the normal index range.

Sera were collected anonymously, coded with indications of age and gender only. The County Ethical Committee, Ferrara approved the project. Written informed consent was obtained from all participants at the time of the hospital admission. All serum samples were stored at −20°C until testing.

### Synthetic Peptides

**Figure 1:**
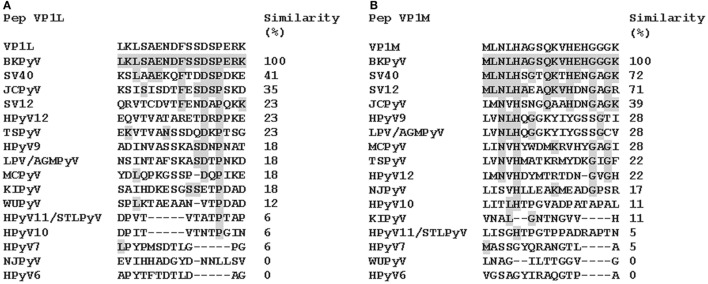
**Similarity among BK polyomavirus (BKPyV)-specific VP1 mimotopes, VP1L (A) and VP1M (B) and other polyomavirus VP1**. Amino acid (a.a.) sequences of two different peptides of viral protein 1 (VP1) of BKPyV. The peptides were selected on the basis of their low homology with the corresponding peptides of other polyomaviruses. Preliminary enzyme-linked immunosorbent assay indicated that only VP1 L and VP1 M peptides reacted with human serum antibodies without cross-reaction with JCPyV and SV40 immune-sera employed as controls. Similarity between synthetic peptides specific to BKPyV and other polyomavirus a.a. sequences: simian virus 40 (SV40), human polyomavirus JC (JCPyV), simian virus 12 (SV12), human polyomavirus 12 (HPyV12), trichodysplasia spinulosa-associated polyomavirus (TSPyV), human polyomavirus 9 (HPyV 9), B-lymphotropic polyomavirus (LPV), Merkel cell polyomavirus (MCPyV), human polyomavirus KI (KIPyV), human polyomavirus WU (WUPyV), Saint Louis polyomavirus (STLPyV), Malawi polyomavirus/human polyomavirus 10 (HPyV10), human polyomavirus 7 (HPyV 7), New Jersey polyomavirus/human polyomavirus 13 (NJPyV), and human polyomavirus 6 (HPyV 6).

Computer-assisted analyses enabled two specific BKPyV peptides to be selected from the late viral region by comparing VP1 proteins from BKPyV, with amino acids from JCPyV and SV40 polyomaviruses, which are highly homologous to BKPyV, as well as with other less homologous polyomaviruses (http://blast.ncbi.nlm.nih.gov; Figure 1; Figure S1 in Supplementary Material). Preliminary ELISA results indicated that the two peptides did not cross-react with the SV40 and JCPyV hyperimmune sera employed (see data in the Section “Results”). The amino acid (a.a.) sequences of the two peptides, known as VP1-L and VP1-M, which start at the N-terminal with a.a. L and M, respectively, are as follows:

VP1-L: NH2—LKLSAENDFSSDSPERK—COOH
VP1-M: NH2—MLNLHAGSQKVHEHGGGK—COOH

Amino acid sequences of BKPyV viral capsid protein 1 (VP1), named peptide L viral capsid protein 1 (VP1 L) and VP1 peptide M (VP1 M), were characterized regarding stable secondary structure formation. Analysis was carried out by PSIPRED server (21–23). Moreover, peptide sequences were mapped on native virion proteins to verify structural similarities. VP1-L and VP1-M, in their monomeric forms, were obtained from computational prediction carried out by I-TASSER server (24–26). Molecular visualizations were performed by PyMOL (PyMOL Molecular Graphics System, Version 1.3, Schrödinger, LLC). Computational tools were available through ExPASy server (27).

### Indirect ELISA

Indirect ELISA was developed and standardized to detect specific antibodies against BKPyV VP1 in human sera using VP1-L and VP1-M synthetic peptides.

#### Peptide Coating

Plates were coated with 5 μg of the selected peptide for each well and diluted in 100 μl of Coating Buffer (Candor Bioscience, Wangen im Allgäu, Germany) at 4°C for 16 h.

#### Peptide Blocking

Blocking was made with 200 μl/well of the Blocking Solution (Candor Bioscience, Germany) at 37°C for 90 min.

#### Primary Antibody Adding

Wells were covered with 100 μl of serum sample diluted 1/20 in low cross-buffer (Candor Bioscience, Germany). Different wells were covered with different kind of serum samples: (1) the BKPyV positive-control, represented by immune rabbit serum containing anti-BKPyV antibodies; (2) the negative controls represented by immune sera anti-SV40 and anti-JCPyV; (3) human serum samples, which were found to be BKPyV-negative in previous investigation using the hemagglutination inhibition (HAI) assay. Each sample was analyzed three times in duplicate wells.

#### Secondary Antibody Adding

The solution contained a goat anti-human or anti-rabbit IgG heavy and light chain specific peroxidase-conjugate (Calbiochem-Merck, Darmstadt, Germany) diluted 1:10,000 in low cross-buffer.

#### Dye Treatment and Spectrophotometric Reading

Samples were treated with 100 μl of 2,2′-azino-bis 3-ethylbenzthiazoline-6-sulfonic acid solution (Sigma-Aldrich, Milan, Italy), for 45 min. at RT and then read on the spectrophotometer (Thermo Electron Corporation, model Multiskan EX, Helsinki, Finland) at a wavelength (λ) of 405 nm. The color intensity in wells where the immunocomplexes were formed was determined by optical density (OD).

#### Cutoff Determination

Cutoff values were determined for each assay using the OD reading of the three negative control sera that were added to the SD and multiplied three times (+3SD). The three BKPyV negative control sera were selected from those below the cutoff value determined with second-degree polynomial regression by plotting the ranked net OD individual values for each peptide. A tendency curve was drawn from a second-degree polynomial regression for VP1 L and M peptides, as published before for MCPyV and BKPyV VLP (28, 29).

Sera with antibodies against BKPyV were considered VP-positive upon reacting to both peptides of the late region and when sera that had been analyzed three times by indirect ELISA testing gave the same positive results.

### BKPyV Hemagglutination (HA) and HAI Assays

A BKPyV viral working stock, Gardner strain (1, 19), for the HAI assay was obtained from infected Vero cells (African green monkey kidney cells, ATCC catalog number CRL-1587) as described before (19, 30, 31). The viral titer, determined by HA assay, was 1.6 × 10^2^ hemagglutinating units, corresponding to 1.6 × 10^6^ plaque-forming units (PFUs)/ml (19). BKPyV was employed as antigen in both HA and HAI assays.

BK polyomavirus HA and HAI titrations were carried out as described in detail elsewhere (19, 30, 31). The HA titer was calculated based on the highest virus dilution that gave complete HA. The HAI assay allows the detection of serum antibodies against the polyomavirus BKPyV, which abolish its agglutination property. The HAI titer was defined as the highest dilution of each serum sample that inhibited viral HA completely.

### BKPyV Neutralization Assay with Human Serum Samples

Permissive Vero cells were used as described and modified slightly as follows: cells were grown and propagated in BioWhittaker^®^ RPMI 1640 with l-glutamine medium (RPMI), supplemented with 10% fetal bovine serum (FBS) at 37°C in a humidified atmosphere with 5% CO_2_ (19). BKPyV infectivity neutralization was carried out by incubating each human serum, diluted at 1:20 in phosphate-buffered saline (PBS) with 5 × 10^6^ PFU of BKPyV at 37°C for 30 min. Then, the suspension was added to Vero cell monolayers for 2 h at 37°C. The inoculum was removed, cells were washed three times with RPMI, and overlaid with the medium containing 1% FBS. Infected Vero cell monolayers were maintained in RPMI + 1% FBS for 3 weeks, until cytopathic effect (CPE) appearance (19, 32). Each sample was tested in duplicate and with different sera with distinct levels of antibodies (OD reading). The neutralization assay included the following controls (i) BKPyV only in PBS and (ii) cells only in PBS. Cultures were observed using a light microscope for CPE presence for 3 weeks.

BK polyomavirus CPE was assessed by three cell biologists of our working group. CPE inhibition was graded as negative, grade 0 (complete CPE), grade 1–2 as weak inhibition, grade 3 as moderate inhibition, grade 4 as intermediate inhibition, and grade 5 as strong inhibition.

### Statistics

Differences among age groups were statistically evaluated. Statistical analyses were performed using Prism 4.0 software (GraphPad, San Diego, CA, USA). Data are presented as a percentage of the positive samples. The 95% confidence intervals of the percentage of positive samples are also reported. Differences among proportions were calculated by χ^2^ test for independence in the contingency tables. The small sample size was statistically analyzed using χ^2^ with Yates’ correction. The correlation between the OD value and BKPyV CPE was evaluated by non-parametric Spearman analysis, which indicates Spearman *r* and *p* values.

The serologic profile of serum antibody reactivity were analyzed with one way Anova analysis, and Newman–Keuls Multiple Comparison Test (OD mean, 95% CI).

## Results

In this study, a novel indirect ELISA with synthetic peptides as antigens was developed and set up to analyze the prevalence of serum antibodies against BKPyV in healthy adult subjects. Two peptides were selected from the amino acids in the VP1 L (VP1), which mimic unique BKPyV VP1 epitopes/antigens (Figure 1; Figure S1 in Supplementary Material). Computational analysis of the two peptides is characterized by their secondary and tertiary structures. Serum samples from HS were analyzed using the new indirect ELISA to detect specific IgG serum antibodies against BKPyV. BKPyV titers were determined by serial sera dilution, which was then analyzed again using indirect ELISA. Immunologic results were compared to data obtained from sera analyzed by the well-established HAI assay (19, 30), employed as control. Furthermore, serum samples, which tested BKPyV-positive, were functionally assayed by inhibiting the viral CPE displayed in the permissive Vero cell line (19).

### Peptide *In Silico* Structural Analysis

**Figure 2:**
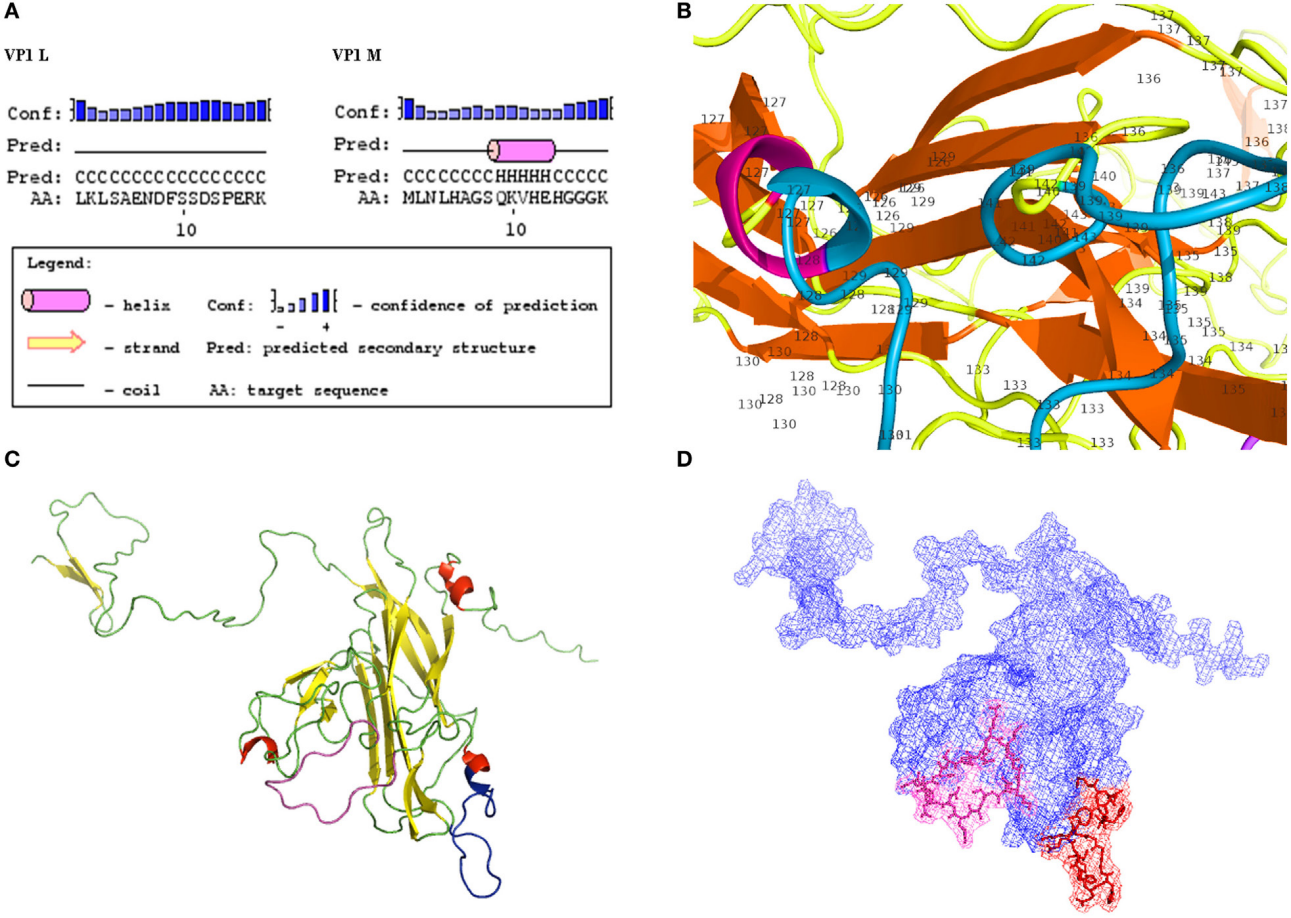
**Structural characteristics of VP1**. **(A)** Secondary structures. Results of computational analysis carried out on BK polyomavirus (BKPyV) synthetic polypeptides (PSIPRED). Peptide VP1L, on the left, shows a random coiled domain, whereas peptide VP1M, on the right, contains an alpha helix domain. **(B)** Detailed view of VP1M peptide (blue) within the three-dimensional (3D) model of BKPyV VP1. Alpha helix domain is shifted to a.a. 126 and a.a. 127, being part of alpha helix structure (red) of the native protein. **(C–D)** 3D model of BK VP1 capsid protein. **(C)** VP1 L (random coiled) and VP1 M (small alpha helix domain) synthetic peptides are shown in pink and blue, respectively. **(D)** Mesh surface model of BKPyV VP1 with VP1L (pink) and VP1M (red) mapped in it. The peptide structures and their localization on the surface of the native protein are marked in pink (L) and red (M), respectively.

Computational analysis showed that the two linear peptides (http://blast.ncbi.nlm.nih.gov; Figure 1; Figure S1 in Supplementary Material) are characterized by their secondary structure, as follows (PSIPRED server). Peptide VP1-L has a random coil secondary structure, whereas it does not contain any alpha helix or beta sheet domain (Figure 2A, on the left). Peptide VP1-M forms a stable secondary structure from a.a. 9 to a.a. 13, i.e., _9_QKVHE_13_, where an alpha helix domain is present (Figure 2A, on the right).

Tertiary structures of BKPyV viral capsid protein VP1 (Figures 2B–D), selected among computationally determined structures, presented *C*-scores of 0.51, with an estimated model accuracy: 0.78 ± 0.10 (TM-score), 5.5 ± 3.5Å (RMSD). For details, see Ref. (24–26). Mapping two linear peptides of the inferred protein structures showed that VP1-L does not fold in any structure type, whereas VP1-M folds in a short alpha helix domain (Figures 2B,C). The three-dimensional graph from L and M peptide structures, shows the specific amino acids exposed to the protein surface (Figure 2D), which may interact with the environment.

### BKPyV Antibodies Detection by Indirect ELISA

An indirect ELISA was setup using synthetic peptides in order to check whether human sera contain IgG antibodies, which react to BKPyV peptides and to determine the spread of BKPyV infection in humans. These synthetic peptides correspond to BKPyV VP1 mimotopes. The unrelated human peptide hNPS was employed as a negative control (19).

In the first step of this investigation, indirect ELISA was used to test serum samples taken from HS ranging from 18 to 90 years old. The sera were diluted at 1/20 in order to react to BKPyV VP1 epitopes. Serum samples, which reacted to BKPyV VP1-L mimotope reached an overall prevalence of 80%. The same assay was then performed to detect IgG class serum antibodies against BKPyV VP1 epitopes, which are known as VP1-M. It turned out that serum samples reacted with the VP1 M peptide with the same prevalence, 80%, as had been detected previously for the VP1 L peptide. Conversely, seronegative samples for the BKPyV VP1 L peptide failed to react with BKPyV VP1 M epitopes. The exceptions were negligible and were represented by a few serum samples, which were found to be negative for VP1 L, while testing positive for VP1 M peptide, and vice-versa. The difference was not statistically significant (*P* > 0.05) (Table 1).

**Table 1:** **Prevalence of immunoglobulin G antibodies reacting to BK polyomavirus (BKPyV) viral protein mimotopes**.

Age (years)	Number of samples	Male (%)	Number of positive samples (prevalence %)		
			VP1-L	VP1-M	VP1 L + M
18–39	118	28	101 (86)	95 (80)	90 (76)
40–50	120	35	107 (89)	103 (86)	97 (81)
51–61	105	49	80 (76)	76 (72)	68 (65)
62–90	103	41	71 (69)	82 (73)	67 (65)
18–90	446	37	359 (80)	356 (80)	322 (72)

*Human sera were from healthy adult subjects. Statistical analysis was performed using the chi-square test with Yates’ correction. The prevalence of BKPyV antibodies detected in the cohort of individuals 51–61 years old is statistically different from that revealed in the cohorts of subjects 18–39 years old (*P* = 0.02) and 40–50 years old (*P* = 0.01), respectively. The prevalence of BKPyV antibodies in the cohort of individuals 62–90 years old is statistically different compared to that of the cohorts 18–39 years old (*P* = 0.018) and 40–50 years old (*P* = 0.008), respectively. Human sera were diluted 1/20. OD = 0.19 was used as a cutoff to identify BKPyV-positive samples. No different prevalence of BKPyV-positive samples was detected between male and female sera (*P* > 0.05)*.

Combining the BKPyV-positive sera, both for the VP1 L and VP1 M peptides, the overall prevalence was 72%, with no difference between male and female (Table 1). The two indirect ELISA tests, with two distinct VP peptides gave overlapping results, thus confirming the presence of anti-BKPyV VP antibodies in human sera from HS (Table 1).

A prevalence selection corresponding to 76, 81, 65, and 65%, within the cohorts ranging from 18 to 90 years old, was observed in subjects 18–39, 40–50, 51–61, and 62–90 years old, respectively. Interestingly, serum antibodies prevalence against BKPyV VP declined in cohorts of individuals ranging from 51 to 61 and 62 to 90 years old, with a percentage of 65% (Table 1).

BK polyomavirus-positive sera tested by indirect ELISA diluted at 1/20 had a general cutoff, range of 0.19 OD, using a spectrophotometric reading. This cutoff represents the discriminatory value between BKPyV-negative samples (below OD 0.19) and BKPyV-positive samples (above OD 0.19). The positive control, represented by BKPyV hyperimmune rabbit serum, had an OD of up to 0.3, while the two JCPyV and SV40 hyperimmune sera, employed as negative controls, had an OD of less than 0.01–0.02. The OD value was usually in the 0.01–0.02 range, which is consistent with the OD for BKPyV-negative sera.

The human neuropeptide hNPS (33), which is unrelated to BKPyV, was employed as a negative control peptide in the ELISA experiments. Data indicate that this negative control peptide did not react with 322 BKPyV-positive sera, nor with 124 BKPyV-negative samples. The OD value was usually in the 0.01–0.02 range, which is consistent with the OD for BKPyV-negative sera.

**Figure 3:**
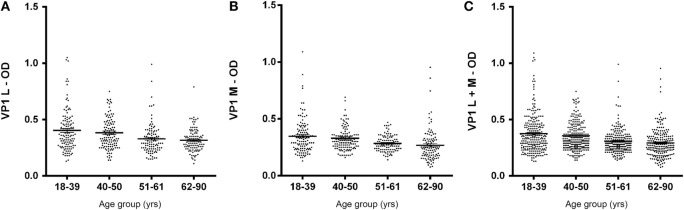
**Serologic profile of human serum antibody reactivity to BKPyV mimotopes VP1 L (A), VP1 M (B), and VP1 L + M (C)**. Immunologic data are from serum samples from HS. Results are presented as values of optical density (OD) readings at λ 405 nm of serum samples diluted at 1:20, detected by the indirect enzyme-linked immunosorbent assay. In scatter dot plotting, each plot represents the dispersion of OD values to a mean level indicated by the line inside the scatter with SEM for each group of subjects analyzed.

In this investigation, BKPyV-positive sera (65%), which was detected both in 51- to 61 and 62- to 90-year-old subjects, was lower than that detected in the other cohorts of healthy individuals (Table 1). These differences are statistically significant when compared to other cohorts from different age groups (18–39; 40–50 years old) (Table 1). The reduced prevalence of BKPyV-positive sera in individuals 51–90 years old could be ascribed to natural age-dependent partial immune decline. Indeed, it is well established that the immune system physiologically declines with age, rendering individuals less responsive to infection. An alternative interpretation of the result, as suggested by other studies (34, 35) is that the low prevalence of BKPyV antibodies in sera from the elderly could depend on low BKPyV antibody stability overtime. Serologic profiles of serum antibody reactivity to BKPyV mimotopes are presented in Figure 3. Serologic profile of serum antibody reactivity to BKPyV mimotopes VP1 L (A) and VP1 M (B) and VPs L + M (C). Data are OD values at 405 nm of serum samples diluted 1:20, detected in indirect ELISA. In scatter dot plotting, each plot represents the dispersion of OD values to a mean level indicated by the line inside the scatter with SEM for each age group of subjects analyzed (mean OD + SEM). The difference in OD mean values were not statistically significant in sera from HSs (*P* > 0.05; Anova analysis, and Newman–Keuls Multiple Comparison Test).

### Serum Antibody Prevalence against BKPyV Determined by HAI Assay

A fraction of randomly chosen human sera (*n* = 87), which had been investigated by indirect ELISA were also analyzed using the well-established technique, known as HAI assay (19, 20, 30). Serum samples, serially twofold diluted from 1:16, 1:32, 1:64, to 1:128, were analyzed by HAI to evaluate both the presence of antibodies against BKPyV and their titer.

The 1:128 dilution was chosen since other lower dilutions, such as 1:32 or 1:64 contain antibodies at higher concentrations, which may give rise to false positive reaction; on the other hand, higher dilutions could carry antibodies at much lower concentration giving false negative reaction (19, 20, 30). The seroprevalence of BKPyV-positive samples, diluted at 1:128, was 63% (55/87). Seropositivity obtained with indirect ELISA on the same sera was 62% (54/87) (Table 2). The seroprevalence of BKPyV-positive in sera from HS, studied by HAI, did not differ statistically from that determined by indirect ELISA (*P* > 0.05). Interestingly, BKPyV-positive samples concordance from the two techniques was 98%.

**Table 2:** **Prevalence of serum IgG antibodies against BK polyomavirus**.

Number of subject	Median age ± SD	Male (%)	Number of positive samples (%) at the dilution indicated			
			1:16	1:32	1:64	1:128
87	69 ± 13	41	85 (98)	85 (98)	84 (97)	55 (63)

*Analyzed using hemagglutination inhibition assay. Human sera were from healthy subjects. The dilution 1:128 has been chosen because lower dilutions, such as 1:32 or 1:64, contain antibodies at higher concentration, which may give rise to false positive reaction; on the other hand, higher dilutions could carry antibodies at much lower concentration giving false negative reaction (19, 30)*.

### Functional Analysis of Serum Antibodies by Neutralization Activity Tested against BKPyV Infection

**Figure 4:**
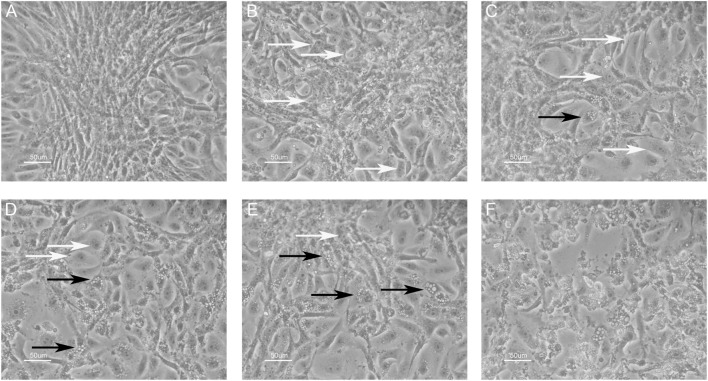
**Inhibition of BK polyomavirus (BKPyV) cytopathic effect (CPE) in infected Vero cells by human serum samples**. White arrows point normal cells, whereas black arrows indicate infected cells. CPE is characterized by the appearance of many vacuoles in the cytoplasm of infected Vero cells in the first phase, whereas the same cells are lysed and destroyed in the final step. **(A)** This panel represents (i) the positive control, i.e., uninfected Vero cells and (ii) the total CPE inhibition. In this image, single normal cells in the periphery and a cluster of them in the middle are shown. **(A–E)** Different degrees of CPE inhibition; there was a correlation between the optical density (OD) reading and the CPE inhibition activity of tested immune sera; the OD level was 0.65, 0.55, 0.45, 0.35, and 0.25, respectively. Sera with lower OD values inhibited less efficiently BKPyV CPE [from **(A–E)**]. Sera with mean OD values 0.65 inhibited BKPyV CPE completely **(A)**, whereas serum samples with mean OD 0.15, BKPyV-negative samples show no CPE inhibition activity **(F)**. The same **(F)** represents the negative control, i.e., BKPyV CPE in infected Vero cells. In this image, many cells show cytoplasmic vacuoles, while in the middle cells are lysed with the loss of the monolayer. Magnification 200x.

**Figure 5:**
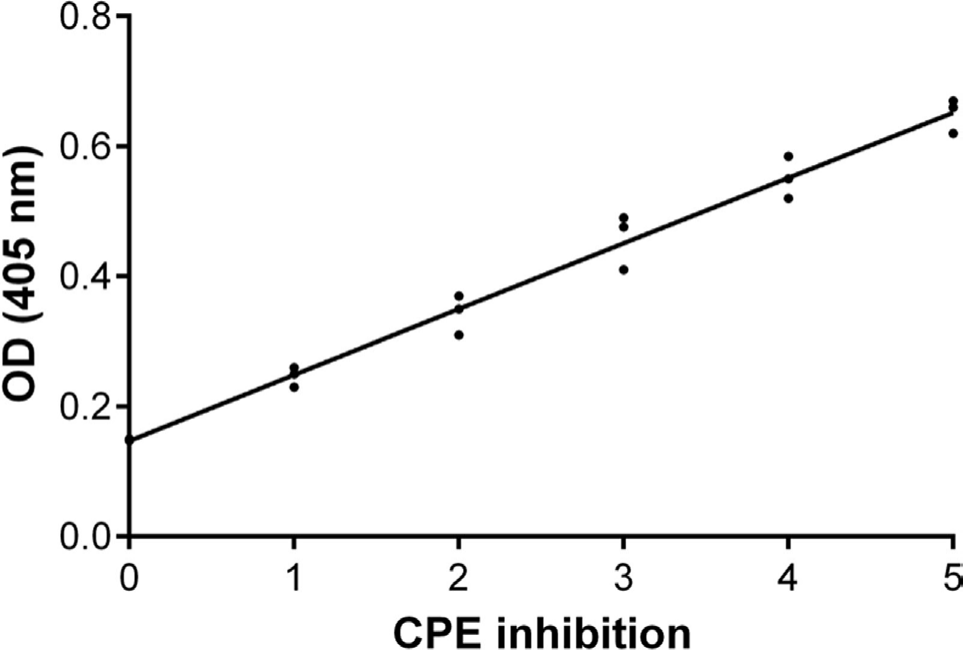
**Inhibition of BK polyomavirus (BKPyV) cytopathic effect (CPE)**. Graph of the functional analysis of BKPyV human immune sera with neutralization activity. Immune sera with higher OD (*Y*-axis) inhibited more efficiently BKPyV CPE (*X*-axis) in infected Vero cell monolayers. Non-parametric correlation Spearman analysis was performed: Spearman *r* = 0.988 and *P* < 0.0001.

An inhibition test was performed in order to check whether immune sera, containing antibodies against BKPyV, neutralized its infectivity. To this end, 15 immune sera, which ranged between a 0.19 and 0.65 OD reading, out of 322 BKPyV-positive samples, were selected to test their ability to inhibit the CPE in permissive BKPyV-infected Vero cells, along with 4 out 124 BKPyV-negative sera, with an OD of below 0.19, employed as control. In this context, it is worth recalling that BKPyV CPE generates many cytoplasmic vacuoles in the first phase of the infection and then cell lysis of Vero monolayers (19). To avoid any possible cross-reactivity, the 15 BKPyV-positive sera analyzed for their neutralization activity, were selected among SV40- and JCPyV-negative sera, as determined by indirect ELISA for SV40 VP (19) and HAI assay for JCPyV (unpublished results). The data are in agreement with previous reports. JCPyV- and SV40-positive sera showed no higher OD values toward VP1 L and VP1 M peptides than those, which tested negative. The neutralization effect of BKPyV-immune sera is apparent in infected cells when BKPyV CPE is abolished or hampered. The CPE inhibition test was performed by mixing the serum sample with BKPyV virions. Then, the mixture was employed as a viral inoculum to infect Vero cells. In inhibition experiments on BKPyV CPE, the positive and negative controls were (i) cells cultured without human serum and without BKPyV and (ii) cells infected with BKPyV, cultured without human serum, respectively. CPE inhibition in BKPyV-infected cells indicated that tested sera were able to neutralize BKPyV infectivity. Indeed, three sera with a mean OD = 0.65, taken from BKPyV-positive samples, completely inhibited BKPyV CPE (Figure 4A), while 12 sera with a mean OD = 0.25–0.55, only partially inhibited BKPyV CPE, with a different degree (Figures 4B–E). Four samples with an OD < 0.19 did not inhibit BKPyV CPE (Figure 4F). There was a correlation between the OD reading and the CPE inhibition activity of the immune sera. Indeed, the correlation between OD and CPE inhibition is statistically significant (Spearman *r* = 0.988 and *P* < 0.0001) (Figure 5).

This assay detects functional antibodies to VP1 L that are capable of neutralizing BKPyV infectivity in tissue culture.

## Discussion

Computational analysis of the two linear peptides VP1-L and VP1-M secondary structure and BKPyV VP1 tertiary structure revealed that VP1-L and VP1-M have secondary structures, which are similar to those found in the containing native protein, although the VP1-M alpha helix (_9_QKVHE_13_) domain is shifted to a shorter NH_2_-terminal alpha helix domain (a.a. 126–127) (Figure 2). The three dimensional mesh surface visualization (Figure 2) of the inferred protein tertiary structure, where the two linear peptides are mapped, showed that analyzed amino acid chains are exposed to the environment. These results may suggest that the two linear peptides VP1-L and VP1-M could resemble natural BKPyV linear epitopes constituting a docking site for serum antibody.

The three polyomaviruses BKPyV, JCPyV, and SV40 show a great a.a. sequence homology. Indeed, protein homology is 72% between BKPyV and JCPyV, 69% between BKPyV and SV40, and 68% between JCPyV and SV40 (6, 19), respectively. High VLPs/rVP1 cross-reactivity observed in previous immunologic tests, prompted us to develop and set up a BKPyV-specific indirect ELISA, with synthetic peptides as antigens, instead of VLP or soluble recombinant VP1.

Herein, human sera were analyzed for IgG antibodies reacting in indirect ELISA to two BKPyV VP1 mimotopes, VP1 L, and VP1 M. As a reaction control, hNPS, an unrelated human synthetic peptide, was employed as a negative control (19).

The objective of the present study was to investigate whether serum samples from HS ranging 18–90 years old carry BKPyV antibodies. Sera were analyzed using two different methods, an indirect ELISA with two mimotopes corresponding to VP1 antigens and HAI assay (19). The two methods were employed to assay the specificity of tests and to confirm data obtained using the innovative ELISA with synthetic peptides as mimotopes of viral capsid proteins 1 (VP1).

These data indicate that the overall prevalence of BKPyV-VP1 antibodies in humans, ranging from 18 to 90 years old, is 72%. No positive results were obtained with the human peptide hNPS used as a control (19).

In the second step of this investigation, human sera from HS were analyzed as control using the well-established HAI assay. This assay allows to detect serum antibodies against polyomavirus BKPyV, which abolish its agglutination property. The seroprevalence of BKPyV-positive samples, diluted 1:128, was 63% (55/87) in HS. HAI data were compared to those obtained by indirect ELISA. The seroprevalence of BKPyV-positive samples from HS determined by HAI does not significantly differ from that obtained using the indirect ELISA with synthetic peptides corresponding to VP1 epitopes (*P* > 0.05) (Table 1).

A neutralization activity test was then carried out as a functional analysis on BKPyV-positive sera. It is important to note that BKPyV prevalence in human sera, as detected by this immunological study, does not differ substantially from that obtained by using neutralization assay against BKPyV infectivity, which is considered the gold standard for measuring BKPyV antibodies in terms of neutralization activity (19).

When considered overall, these ELISA data indicate that, in normal individuals, natural BKPyV infection occurs at a high prevalence (72%), when compared with the low prevalence of infection spread over other new polyomaviruses, which range from 20 to 50% (36). The prevalence of anti-BKPyV serum antibodies increases with age, reaching 81% in the cohort from 40 to 50 years old, with an increase of BKPyV antibody levels determined by OD readings.

It has been shown that BKPyV is present in the urine, stool, upper respiratory tract, and blood specimens in HS suggesting that different transmission routes are responsible for BKPyV infection.

Functional analysis data on BKPyV infectivity inhibition obtained with immune sera from normal subjects are of interest. There is a correlation between the grade of BKPyV infection inhibitory effect by immune sera and antibody level of as determined by OD readings. A higher concentration of serum antibodies gave a stronger inhibition effect on BKPyV infection in tissue cultures.

Since the selected BKPyV-immune sera were JCPyV- and SV40-negative, there is no possible cross-inhibition effect due to antibodies against the two closely related polyomaviruses. Indeed, BKPyV and JCPyV are ubiquitous in the human population with a prevalence of up to 90 and 60%, respectively (2, 6), whereas SV40-positive sera account for approximately 20% of human samples (19).

In order to further examine BKPyV-positive samples serology, endpoint titers were determined using indirect ELISA. The highest endpoint titer was observed at a 1/160 dilution for both VP 1L and VP1 M peptides.

In conclusion, the innovative indirect ELISA with BKPyV VP1 mimotopes reported herein seems to be a useful new method for detecting specific IgG antibodies against this virus in human sera, without cross-reactivity with other small DNA tumor viruses, such as the closely related SV40 and JCPyV polyomaviruses.

## Ethics Statement

This study was carried out in accordance with the recommendations of the County Ethics Committee of Ferrara with written informed consent from all subjects. All subjects gave written informed consent in accordance with the Declaration of Helsinki. The protocol was approved by the County Ethics Committee of Ferrara.

## Author Contributions

FM and MT conceived and designed the experiments. GG and PN provided clinical samples. SP, FL, and AP performed the experiments. MM and PN analyzed the data. FM, SP, IB, and MT wrote the paper. FM, EM, FD, and MT critically revised the manuscript. All authors read and approved the final manuscript.

## Conflict of Interest Statement

Data of this work were enclosed, in part, in the Italian patent application number I0167478/BRE-EC/rp, filed on August 9, 2016.
