# Supplementary material for: Specific IgG Antibodies React to Mimotopes of BK Polyomavirus, a Small DNA Tumor Virus, in Healthy Adult Sera
Source: Front Immunol. 2017 Mar 6;8:236. doi: 10.3389/fimmu.2017.00236 (PMC5338004; doi:10.3389/fimmu.2017.00236)
Supplement: Supplementary file 1 [file Image_1.PDF]

SUPPLEMENTARY MATERIAL

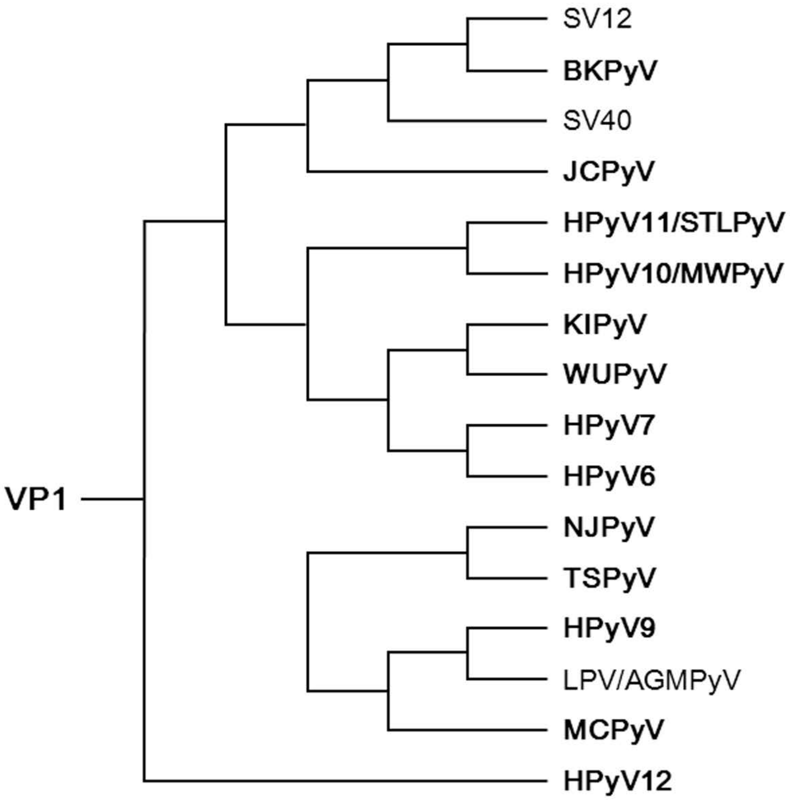

FIGURE S1. Polyomavirus phylogenetic tree based on VP 1 a.a. sequences. Representation of the polyomaviruses phylogenetic tree. The similarity of VP 1 a.a. sequences among different polyomaviruses is shown. BKV (BKPyV, BK polyomavirus) VP 1 is more closely related to those of SV40 (Simian Virus 40), SV12 (Simian Virus 12) and JCV (JCPyV, JC Polyomavirus) than to the VP1 of other polyomaviruses: KIV (KIPyV, KI Polyomavirus), WUV (WUPyV, WU Polyomavirus), HPyV11/STLPyV (Human Polyomavirus 11), HPyV10 (Human Polyomavirus 10), HPyV7 (Human Polyomavirus 7), HPyV6 (Human Polyomavirus 6), HPyV9 (Human Polyomavirus 9), LPV/AGMPyV (B-lymphotropic Polyomavirus), TSV (TSPyV, Trichodysplasia spinulosa-associated polyomavirus), MCPyV (Merkel cell polyomavirus), HPyV12 (human polyomavirus 12), and NJPyV (New Jersey polyomavirus, not shown).
